# Supplementary material for: Independent Negative Prognostic Role of TCF1 Expression within the Wnt/β-Catenin Signaling Pathway in Primary Breast Cancer Patients
Source: Cancers (Basel). 2019 Jul 22;11(7):1035. doi: 10.3390/cancers11071035 (PMC6678184; doi:10.3390/cancers11071035)
Supplement: Supplementary file 1 [file cancers-11-01035-s001.pdf]

Supplementary Materials

Table S1. Tumor characteristics of 220 invasive breast cancer patients.

Characteristics	n	(%)
Patients Age: median value 54 (range 28–80)		
≤54 years	114	(51.8)
>54 years	106	(48.2)
Histological type		
IDC	187	(85)
ILC	18	(8.2)
Other	15	(6.8)
Histological grade		
G1-2	121	(55)
G3	93	(42.3)
Unknown	6	(2.7)
Tumor size (cm)		
≤2 cm	130	(59.1)
>2 cm	81	(36.8)
Unknown	9	(4.1)
Lymph node status		
Negative	129	(58.6)
Positive	85	(38.6)
Unknown	6	(2.7)
Estrogen receptor		
ER-negative (≤10%)	66	(30)
ER-positive (>10%)	150	(68.2)
Unknown	4	(1.8)
Progesterone receptor		
PR-negative (≤10%)	97	(44.1)
PR-positive (>10%)	119	(54.1)
Unknown	4	(1.8)
Ki67		
Negative (≤20%)	126	(57.3)
Positive (>20%)	89	(40.4)
Unknown	5	(2.3)
HER2/neu		
Negative (0,1+)	185	(84.1)
Positive (3+)	28	(12.7)
Unknown	7	(3.2)

ER: Estrogen receptor; PR: Progesterone receptor; HER2/neu: Human epidermal growth factor receptor 2.
